# New Silurian aculiferan fossils reveal complex early history of Mollusca

**DOI:** 10.1038/s41586-024-08312-0

**Published:** 2025-01-08

**Authors:** Mark D. Sutton, Julia D. Sigwart, Derek E. G. Briggs, Pierre Gueriau, Andrew King, David J. Siveter, Derek J. Siveter

**Affiliations:** 1 Department of Earth Science and Engineering, Imperial College, London, UK; 2 Marine Zoology Department, Senckenberg Research Institute and Museum, Frankfurt, Germany; 3 Institute of Ecology, Evolution and Diversity, Goethe University, Frankfurt, Germany; 4 Department of Earth and Planetary Sciences, Yale University, New Haven, CT USA; 5 Yale Peabody Museum, New Haven, CT USA; 6 Université Paris-Saclay, CNRS, Ministère de la Culture, UVSQ, MNHN, IPANEMA UAR3461, Saint-Aubin, France; 7 Institute of Earth Sciences, University of Lausanne, Lausanne, Switzerland; 8 SOLEIL synchrotron, Gif-sur-Yvette, France; 9 School of Geography, Geology and the Environment, University of Leicester, Leicester, UK; 10 Earth Collections, University Museum of Natural History, Oxford, UK; 11 Department of Earth Sciences, University of Oxford, Oxford, UK

**Keywords:** Palaeontology, Phylogenetics

## Abstract

Mollusca is the second most species-rich animal phylum, but the pathways of early molluscan evolution have long been controversial^1–5^. Modern faunas retain only a fraction of the past forms in this hyperdiverse and long-lived group. Recent analyses^6–8^ have consistently recovered a fundamental split into two sister clades, Conchifera (including gastropods, bivalves and cephalopods) and Aculifera^9^, comprising Polyplacophora (‘chitons’) and Aplacophora. Molluscan evolution in toto is characterized by plasticity in body-plan characters^10^, but historically aculiferans have been interpreted as more conservative^10,11^. The few completely preserved aculiferan or aculiferan-like fossils from the early Palaeozoic^12–19^ have been largely regarded as transitional forms that inform questions of character polarity between the extant polyplacophoran and aplacophoran body forms^20,21^. The history of early aculiferans, and the morphological and ecological range that they occupied, remain inadequately sampled. Here we describe two new three-dimensionally preserved aculiferan species from the Silurian Herefordshire Lagerstätte^22,23^, which substantially extend the morphological and ecological range of the clade. Phylogenetic analyses indicate positions within a complex nexus of taxa and suggest reversals in the states of fundamental characters such as the presence of valves and the nature of the foot. In contrast to previous hypotheses of morphological conservatism, evolution in early aculiferans generated a profusion of unusual forms comparable to the diversification of other crown-group molluscs.

## Abstract (web summary)

Fossils of two new worm-like aculiferan species add to the diversity of this group, showing that evolution in early aculiferans generated unusual forms comparable to other crown-group molluscs.

## Main

Aculiferans typically possess a scleritome of cuticular aragonitic spicules^9^. Polyplacophorans are characterized by a series of eight dorsal valves and a ventral foot surrounded by gills. Aplacophorans, comprising the classes Solenogastres and Caudofoveata, have a vermiform shell-less body with a posterior respiratory cavity^10^ and a reduced or absent foot. Living aculiferans were interpreted historically not as a clade but as basal branching groups within Mollusca, because of their relatively simple morphology^11^; chitons, for example, have been labelled ‘living fossils’^24^ because they lack cephalized senses and have conservatism in form throughout the fossil record.

Early Palaeozoic fossils include many ‘chiton-like’ valves^25–34^ as well as sclerite- or spicule-bearing forms that have been variously placed within (or compared with) Aculifera^12–19^. Completely preserved examples are rare, but important in documenting body-plan configurations and character-state combinations outside the scope of the crown-group lineages. The Silurian genus *Acaenoplax*, for example, the most aplacophoran-like early fossil described to date, bears a series of shell valves as well as spicules^14,19^. All complete fossils assigned to the polyplacophoran subclass Paleoloricata have chiton-like valves associated with a vermiform body that lacks a foot but possesses a posterior respiratory cavity^15,18^. The morphological, ecological and temporal range occupied by total-group Aculifera is poorly constrained, yet remains crucial to understanding molluscan body-plan evolution.

We describe two new vermiform aculiferan molluscs, *Punk ferox* and *Emo vorticaudum*, from the Silurian Herefordshire Lagerstätte (approximately 430 million years before present), a deposit that preserves a diverse open marine biota in remarkable detail^22,23^. The specimens were reconstructed three-dimensionally through a combination of physical–optical tomography and phase-contrast synchrotron X-ray microtomography (SXMT). The new genera substantially expand the morphological range of Palaeozoic aculiferans, providing evidence for previously unknown character combinations and locomotion mechanisms.

## Systematic palaeontology

Phylum Mollusca

*Punk ferox* Sutton, Sigwart, Briggs, Gueriau, King, Siveter and Siveter gen. et sp. nov.

**LSID**. *Punk*: urn:lsid:zoobank.org:act:06C4B4EE-8FA2-43B6-9BB0-67DE8816C3A4. *Punk ferox*: urn:lsid:zoobank.org:act:2F594C68-75BB-4E62-B93E-A08BB5753FFC.

**Etymology**. *Punk*: fancied resemblance of the spicule array to the spiked hairstyles associated with the punk rock movement; *ferox* (Latin): wild, bold, defiant. Grammatical gender: nonbinary.

**Fig. 1:**
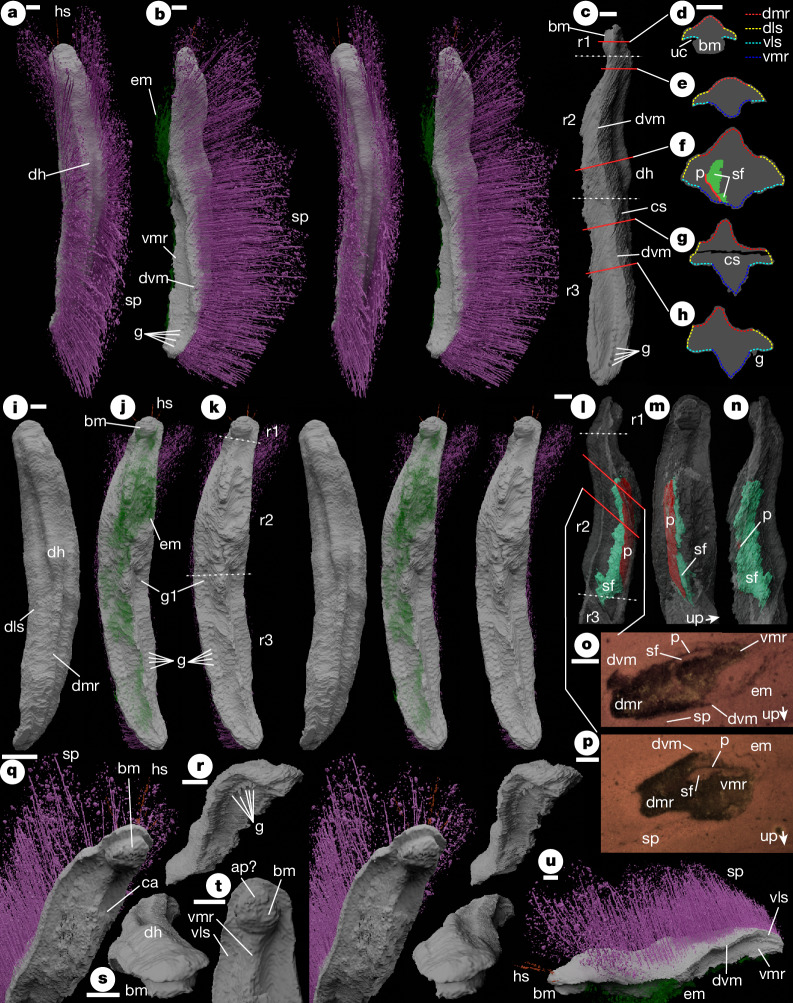
OUMNH PAL-C.29644; holotype of *Punk ferox* gen. et sp. nov. **a**–**c**,**i**–**n**,**q**–**u**, Virtual reconstructions from physical–optical dataset. **d**–**h**, Cross-sections. **o**,**p**, Optical tomograms. **a**, Dorsal stereo-pair. **b**, Left lateral stereo-pair. **c**, Left lateral (without spines and ?extraneous material) showing positions of regions 1–3 (white dotted lines) and cross-sections **d**–**h** (red lines). **d**–**h**, Cross-sections traced from virtual reconstruction with the extent of lateral surfaces and median ridges indicated. All scales are as shown in **d**. **i**, Dorsal stereo-pair (without spines). **j**, Ventral stereo-pair. **k**, Ventral stereo-pair (without spines and ?extraneous material) indicating regions 1–3. **l**, Right lateral of anterior (without spines and ?extraneous material, with body translucent) showing positions of regions 1–3 (white dotted lines) and tomograms **o** and **p** (red lines). **m**, Ventral of anterior (as **l**, scale as **l**). **n**, Left lateral of anterior (as **l**, scale as **l**). **o**,**p**, Optical tomogram Punk_B_PO_212.bmp^41^ (**o**) and Punk_B_PO_152.bmp^41^ (**p**). **q**, Anterior oblique stereo-pair. **r**, Posterior oblique stereo-pair (without spines and ?extraneous material). **s**, Anterior oblique (as **r**). **t**, Ventral detail of anterior (without spines and ?extraneous material). **u**, Left lateral oblique. ap?, putative anterior plate on buccal mass; bm, buccal mass; ca, concavity on left side; cs, concretion split; dh, dorsal ‘hump’; dls, dorsolateral surface; dmr, dorsal median ridge; dvm, dorsoventral margin; em, ?extraneous material; g, gills; g1, first (anterior-most) gill; hs, head spines; p, internal plate; r1–3, regions 1–3; sf, sediment fill; sp, spines; uc, undercut beneath buccal mass; up, younging direction indicated by sediment fill; vls, ventrolateral surface; vmr, ventral median ridge. Scale bars, 1 mm.

**Holotype**. Oxford University Museum of Natural History, OUMNH PAL-C.29644, the only known specimen (Fig. 1).

**Stratigraphy and locality**. Wenlock Series, Silurian; Herefordshire, England.

**Diagnosis for genus and species**. Vermiform, with dorsal and ventral median ridges. Lacking valves. Scleritome of mostly dorsally directed elongate spicules arising dorsolaterally and posteriorly. Ventral tissues non-cuticular. Approximately 25 gill pairs flanking ventral median ridge in posterior half of trunk.

**Description** (expanded in Supplementary Note 1). Trunk elongate and vermiform, rounded at anterior and posterior terminations (Fig. 1a–c,i–k,q–s,u). Dorsal surface with median ridge (Fig. 1i,r,s,u), broader anteriorly than posteriorly, with low ‘hump’ near midpoint (dh; Fig. 1a,i,s). Ridge flanks (concave-up in cross-section) transition laterally into dorsolateral surfaces (typically convex-up in cross-section; Fig. 1d–h); these meet at the posterior extremity of the trunk (Fig. 1r).

Valves absent. Dorsolateral surfaces bear long spines, interpreted as mineralized spicules (Fig. 1a,b,o–q,u). Spine length varies little along trunk except near terminations (Fig. 1b). Spines directed dorsally and weakly recurved towards the midline. Spine array open dorsally, except in trunk posterior (Fig. 1a). Spine array fans out anteriorly and laterally towards the trunk anterior (Fig. 1a,j,k,u). Anterior margin bears several short ‘head’ spines (Fig. 1a,j,q,u).

Dorsal surface better preserved than ventral. Surfaces separated by a sharp margin (dvm; Fig. 1c,o,p,u) differentiating dorsal integument (interpreted as cuticular) from ventral (non-cuticular). Ventral interpretation complicated by the necessarily interpretative boundary between fossil and adherent ‘extraneous material’ (rendered translucent; Fig. 1b,j,u). Ventral morphology described in three regions (r1–3; Fig. 1c,k). Region 1 is a short ‘head’ with subcylindrical ‘boss’ interpreted as buccal mass (Fig. 1c,d,j,k,q,s–u), lacking preserved opening, possibly bearing sub-semicircular anterior plate (Fig. 1t). Ventrolateral surfaces surround buccal mass and continue into region 2 (Fig. 1q,t). Regions 2 and 3 with strong median ridge between subhorizontal lateral regions (Fig. 1b,e–h,k,o–r,t,u), abutting buccal mass anteriorly, varying in cross-sectional profile along trunk. Region 2 extends to near midpoint (Fig. 1c). Asymmetrical ‘inflation’ of median ridge (Fig. 1j,k) may be a post-mortem effect. Posterior boundary of region 2 corresponds to change in width of dorsal median ridge (broader above region 2; Fig. 1c,i,k). Region 3 bears around 25 pairs of short subconical projections adjacent to median ridge, interpreted as gills (Fig. 1b,c,h,j,k,r,u).

Thin plate and geopetal cavity-fill preserved inside ventral median ridge of region 3 (Fig. 1f,l–n,o,p). Plate compositionally differentiated, probably mineralized and preserved displaced from unknown original position.

*Emo vorticaudum* Sutton, Sigwart, Briggs, Gueriau, King, Siveter and Siveter gen. et sp. nov.

**LSID**. *Emo*: urn:lsid:zoobank.org:act:6C11D55C-7C33-4AE4-A848-90A335C39AFF. *Emo vorticaudum*: urn:lsid:zoobank.org:act:8386FD34-6905-42D0-BCF6-DE196510E1EB.

**Etymology**. *Emo*: after the emo musical genre related to punk rock, whose exponents canonically bear long ‘bangs’ or fringes, of which the scleritome is reminiscent, as well as studded clothing recalling the anterior valves; *vorticaudum* (Latin): adjective combining vortex (whirl) and cauda (tail). The name alludes to the rotational twisting of the tail–spine arrangement. Grammatical gender: nonbinary.

**Fig. 2:**
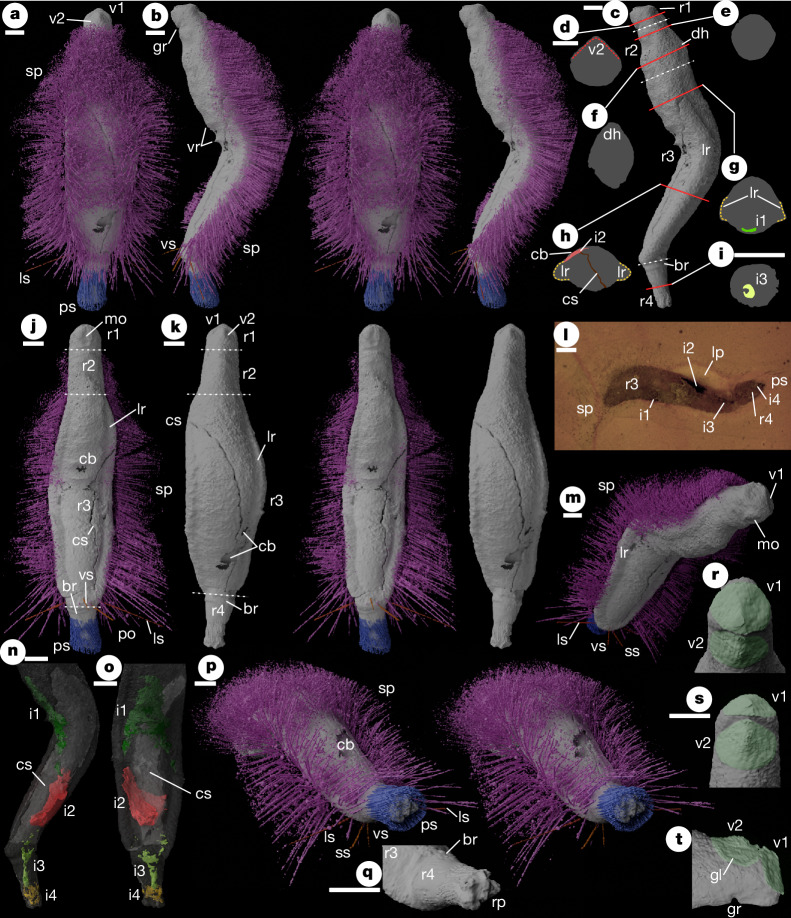
OUMNH PAL-C.36023; holotype of *Emo vorticaudum* gen. et sp. nov. **a**–**c**, **j**,**k**,**m**–**t**, Virtual reconstructions from synchrotron dataset. **d**–**h**, Cross-sections. **l**, Optical tomogram. **a**, Dorsal stereo-pair. **b**, Left lateral stereo-pair. **c**, Left lateral (without spines) showing positions of body divisions (white dotted lines) and cross-sections **d**–**i** (red lines). **d**–**i**, Cross-sections traced from virtual reconstruction. All scales are as shown in **d** (except **i**). **j**, Ventral stereo-pair. **k**, Dorsal stereo-pair (without spines). **l**, Optical tomogram Emo_A_PO_031.bmp^41^, oblique but sublongitudinal, positioned near part-counterpart boundary. **m**, Anteroventral oblique. **n**, Left lateral (missing anterior, without spines, with body translucent). **o**, Ventral (as **n**). **p**, Posterodorsal oblique stereo-pair. **q**, Region 4 (without spines, viewing position as left-hand **p**). **r**, Subanterior, dorsal downwards, looking directly down onto valve I (image manually enhanced to pick out valves in green, for clarity). **s**, Subdorsal, looking directly down onto valve II (as **r**). **t**, Lateral of anterior region (as **r**). br, basal ridge; cb, cuticular break (artefactual); i1–4, internal structures 1–4; gl, growth-line of valve II; gr, groove between regions 1 and 2; lp, light coloured patch in matrix; lr, lateral roll; ls, posterior lateral paired spines; mo, ?mouth; rp, respiratory protuberances/projections; ps, posterior spines; sp, main spine array; ss, ventral submedial spine; v1, valve I; v2, valve II; vr, ventral ridges; vs, postero-ventrally directed spine. Scale bars, 1 mm.

**Extended Data Fig. 1:**
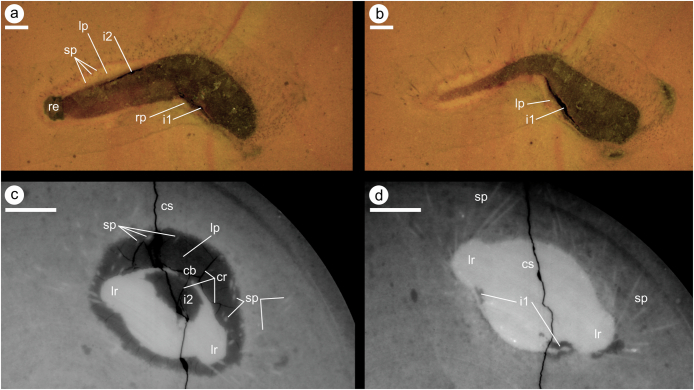
Additional tomograms of *Emo vorticaudum.* OUMNH PAL-C.36023; holotype of *Emo vorticaudum* gen. et sp. nov. **a,b**, Optical tomograms from serial-grinding dataset^41^. **c,d**, Computed tomograms from synchrotron dataset^41^. **a**, File Emo_B_PO_023.bmp, subparallel to part-counterpart boundary, anterior to the right. **b**, File Emo_B_PO_054.bmp, subparallel to part-counterpart boundary, anterior to the right, dorsal up. **c**, File Emo_0911.bmp, subtransverse through middle of ‘bald patch’, dorsal is up/right. **d**, File Emo_1434.bmp, subtransverse slightly posterior to dorsoventral fold, dorsal is up/right. Abbreviations as Fig. 2, with the addition of: cr, cracks; re; opaque resin. Opaque resin is used in physical optical tomography to ensure structures outside the tomographic plane are not imaged^42^; here the resin-covered area represents portions of the plane on the ‘other side’ of the surface along which the concretion split. Scale bars, 1 mm.

**Holotype**. Oxford University Museum of Natural History, OUMNH PAL-C.36023, the only known specimen (Fig. 2 and Extended Data Fig. 1).

**Stratigraphy and locality**. Wenlock Series, Silurian; Herefordshire, England.

**Diagnosis for genus and species**. Vermiform, trunk lacking foot, with rounded convex dorsal and ventral surfaces. Two similar small suboval roof-like valves at anterior. Dorsal and lateral scleritome of spicular spines, absent on the head and ventrally. Articulated posterior region forming respiratory cavity surrounded by posteriorly directed spines.

**Description** (expanded in Supplementary Note 1). Vermiform (Fig. 2c,j,k), weakly dorsoventrally compressed (Fig. 2e–i), preserved with dorsoventral fold near midpoint (Fig. 2b,c). The body comprises four regions (r1–4 anterior to posterior; Fig. 2c,j,k).

Region 1 is a short ‘head’ bearing two similar small valves, Valve I anterior-facing, valve II dorsal-facing (Fig. 2a,k,r–t). Valves in near contact, at a high angle (Fig. 2t). Both valves suboval with distinct and weakly concave lateral areas flanking a median ridge, lacking ornament, but with one growth-line (Fig. 2t). Raised ventromedian region bearing weak elongate median hollow interpreted as the mouth (Fig. 2j,m). Region 1 separated from ventral surface of region 2 by transverse groove (Fig. 2b,t).

Region 2 is a ‘neck’ with near-parallel lateral margins (Fig. 2j,k), expanding dorsoventrally, with pronounced elongate dorsal ‘hump’ (Fig. 2c,f,m,t). Lateral, dorsal and ventral surfaces continuous (Fig. 2e,f,m). Region 3, comprising most of body-length, characterized by lateral rolls, thickenings of lateral margin separating dorsal and ventral surfaces (Fig. 2g,h,j,k,m). Lateral rolls merge anteriorly into lateral surfaces of region 2; posteriorly they meet medially on ventral surface (Fig. 2j,m). Dorsal and ventral surfaces weakly convex (Fig. 2g,h); minor transverse ridges (Fig. 2b) interpreted as in vivo deformation resulting from fold. Dorsal and ventral surfaces of region 3 lacking either preservational differentiation or sharp junction, anteriorly ventral surface continuous with that of region 2, in turn continuous with region 2 lateral and dorsal surfaces. All integument of regions 1–3 thus interpreted as cuticular; foot absent.

Regions 2 and 3 bearing array of dorsal and lateral spines interpreted as mineralized spicules (Fig. 2a,b,j,l,m,p), arising from lateral rolls (the longest spines) and the dorsal surface. Spines absent ventrally and anteriorly. Gap in posterior dorsal scleritome (Fig. 2a,p) interpreted as preservational artefact (Supplementary Note 1). Dorsally directed spines recurved medially; mid-dorsal spines ‘criss-cross’ medially (Fig. 2a,p). Four posterior spines are different in direction to nearby spines (ls, vs, ss; Fig. 2b,j,m,p; Supplementary Note 1).

Region 4 is a ‘tail’, subcircular in cross-section (Fig. 2i), tapering posteriorly from transverse basal ridge (Fig. 2c,j,k,q). The posterior surface bears poorly preserved stubby protuberances, interpreted as respiratory surfaces (Fig. 2q). Region 4 encased in posteriorly directed and rotationally twisted spine array (Fig. 2a,b,j,p) originating near basal ridge, terminating the same distance beyond respiratory projections (Fig. 2p).

Four internal structures (i1–4; Fig. 2g,n,o) are preserved. These are difficult to interpret and may, in part, be artefacts of preservation. Those in region 4 (i3, i4) may represent traces of gut and respiratory structures respectively.

## Discussion

*Punk* and *Emo* are both differentiated dorsoventrally, with spine-free ventral surfaces, implying a vagile epibenthic mode of life. In both cases, however, their locomotory mode must have been unusual. *Punk* possesses the homologue of a foot, but, unlike that in extant molluscs, it forms a ridge-like projection rather than a flat sole. It may have had a locomotory role, but the nature of its interaction with the substrate is unclear. *Emo* possesses a flatter ventral sole, but this was cuticular, so cannot have functioned as a typical molluscan foot. The preservation of the holotype with a dorsoventral median fold may indicate an ‘inching’ locomotive mode, using the relatively robust postero-lateral and postero-ventral spines, separate from the main spicule array, as a ratchet to resist retrograde movement. This locomotion mode would be novel. Inching is currently used by terrestrial caterpillars^35^ and freshwater leeches^36^, both of which facilitate it using anterior adhesion/grip rather than by resistance to posterior motion.

Both *Punk* and *Emo* record new combinations of features including characters long considered to be of paramount importance in molluscan classification and phylogeny. *Punk* resembles aplacophorans in its vermiform body, lack of valves and scleritome of long spines. However, it resembles polyplacophorans in possessing a broad median foot (or foot homologue) with a peripheral respiratory cavity housing serially repeated gills. Restriction of the ventral respiratory cavity to the posterior half of the body is also a feature of the extant plesiomorphic polyplacophoran order Lepidopleurida^37^. *Emo* is similar to caudofoveate aplacophorans^10^ with a broadly vermiform body, an anterior region (region 1) comparable to the caudofoveate anterium, a posterior region (region 4) closely comparable to the posterium of extant caudofoveates^10^, and a dense and complete scleritome of dorsal spines. In addition it lacks a foot and its respiratory structures are posterior not ventral. However, it resembles polyplacophorans in bearing dorsal valves (in the head region), and in being dorsoventrally compressed. Both new genera differ from other Palaeozoic fossils, except for some similarities between *Emo* and the heloplacid *Acaenoplax*^14,19,23^. These include a similar valve morphology and respiratory structures, and the presence of a spine-free cuticular ventral sole. There are, however, significant differences, notably the number of valves and the lack of externally expressed serial repetition in *Emo*.

**Fig. 3:**
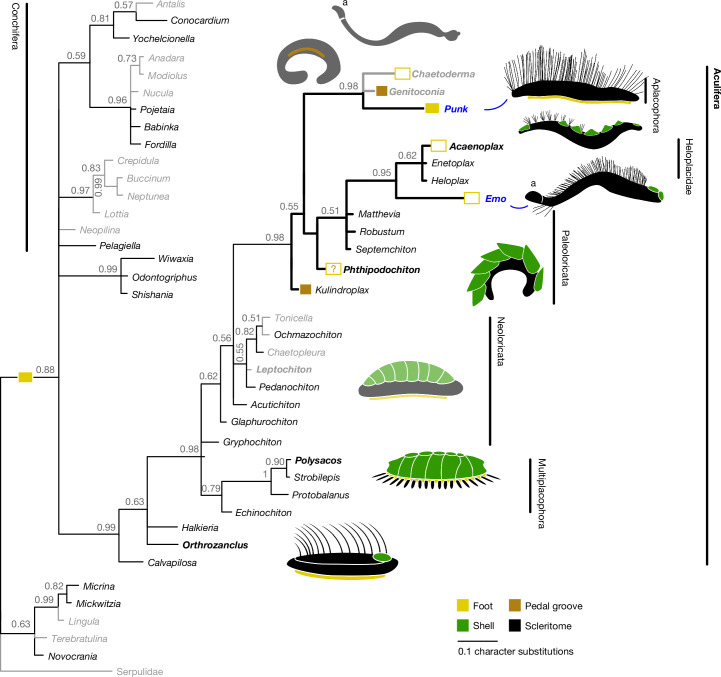
Phylogenetic position of *Punk ferox* gen. et sp. nov. and *Emo vorticaudum* gen. et sp. nov. Majority rule consensus tree of the posterior sample of a Bayesian analysis of fossil and living molluscs based on a morphological matrix of 108 characters. Grey branches are extant; black branches are extinct. Figured taxa are given in bold. Values at nodes are posterior probabilities. Reconstructed foot-character state is given for selected nodes/tips (see Extended Data Fig. 3 for full reconstruction). Branch lengths represent expected number of substitutions per site (indicated by scale bar). a, posterium.

**Extended Data Fig. 2:**
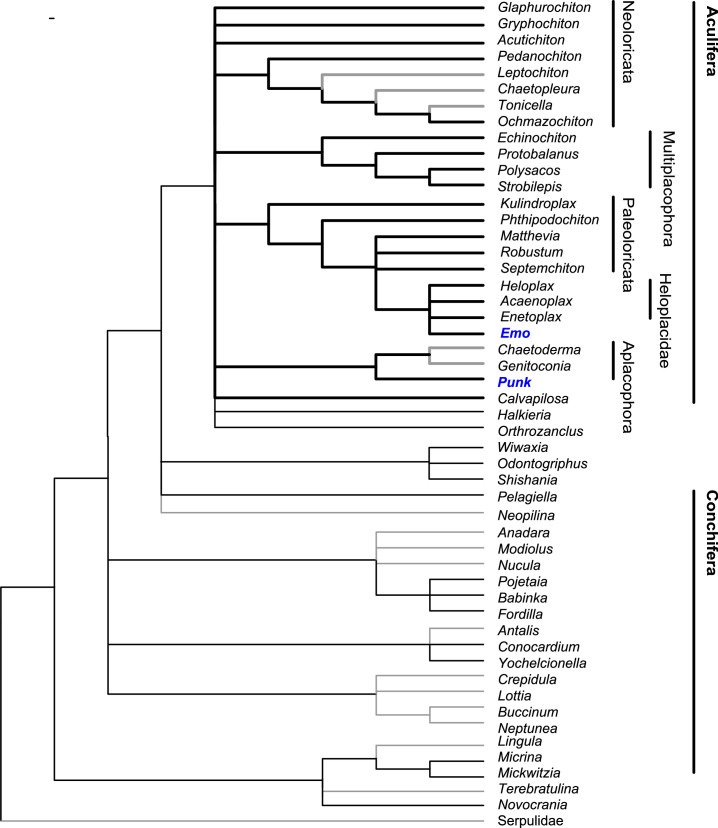
Results of parsimony analysis. Strict consensus cladogram of 1434 trees, length 284, with Serpulidae as outgroup. Grey branches are extant; black branches are extinct.

To elucidate phylogenetic relationships and character-state transitions, we constructed a new morphological character matrix, based on previously published analyses^38,39^ but limiting the matrix to characters relevant to molluscs. Our Bayesian analysis (Fig. 3) recovered a monophyletic Mollusca and Aculifera. Both *Punk* and *Emo* resolved within a broad clade comprising Paleoloricata and Aplacophora, with *Punk* in the same clade as modern Aplacophora, and *Emo* as sister to the heloplacids. The result of the parsimony analysis of the same dataset (Extended Data Fig. 2) was broadly compatible but less resolved, and is not considered further.

In Fig. 3, *Wiwaxia*^13^, *Odontogriphus*^40^ and *Shishania*^39^ resolve as sister taxa within a basal molluscan polytomy in agreement with other analyses^39^; other fossils previously considered as possible aculiferans resolve within the aculiferan clade, with *Halkieria*^12^*, Orthrozanclus*^16^ and *Calvapilosa*^17^ in the stem group. The multiplacophorans^27,33^ (+*Echinochiton*^28^) resolve as a clade sister to crown-group Aculifera, in contrast with previous analyses that placed Multiplacophora as part of total-group Polyplacophora^39^ or stem group Neoloricata^1,15,33^. Extant chitons (plus two neoloricate fossils) form a clade, but the neoloricate chitons *Glaphurochiton*^29^, *Acutichiton*^30^ and *Gryphochiton*^31^ appear as a grade at the base of aculiferan crown group; this may be an artefact reflecting the balance of characters in the analysis and the fragmentary nature of most chiton fossils. All paleoloricate ‘chitons’ in our analysis fall in the aplacophoran stem group. A large clade resolved in this stem contains the clade (*Emo +* Heloplacidae^25^) in addition to three paleoloricate taxa known only from valves (*Septemchiton*^32^*, Robustum*^33^ and *Matthevia*^34^).

**Extended Data Fig. 3:**
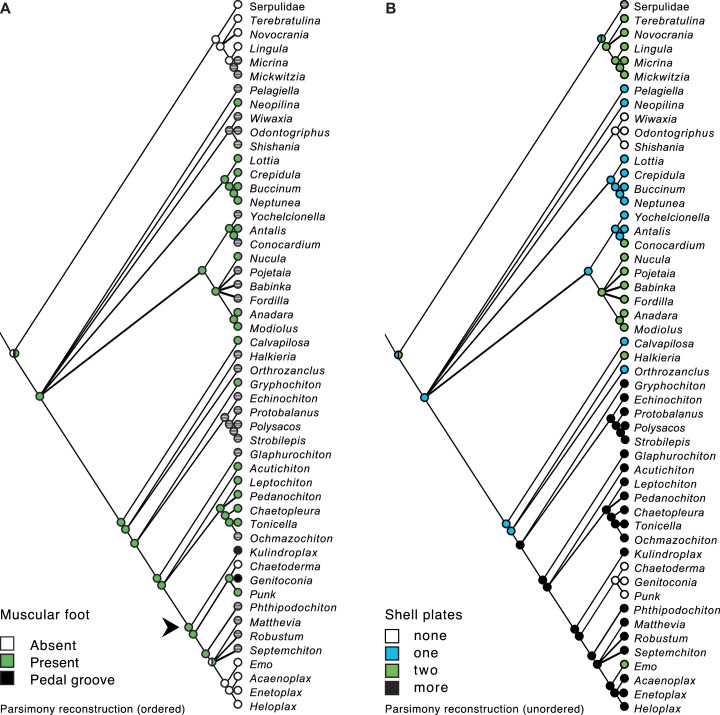
Ancestral state reconstructions for selected characters. A. Presence of a muscular foot (character 16); B. number of shell plates or valves (character 39). Arrowhead denotes total-group Aplacophora (Aplacophora+Paleoloricata).

Ancestral character-state reconstruction (Fig. 3 and Extended Data Fig. 3) recovers convergence in characters that are traditionally considered diagnostic of major groups. Convergent characters include valve count, which is not congruent on any branching-order reconstruction of our tree, and was probably reduced independently in lineages leading to *Punk* + Aplacophora, and to *Emo* (Extended Data Fig. 3). The aplacophoran total-group (including Paleoloricata) is characterized by the absence of a typical foot, but in detail the picture is more complex, with probably convergent acquisition of a pedal groove in solenogastres and *Kulindroplax*, and foot loss occurring in multiple places (Fig. 3 and Extended Data Fig. 3). The aberrant ridge-like foot of *Punk* further complicates the history of this character. Tree-topology also implies that the caudofoveate-like posterium of *Emo* was convergently acquired. These convergences and previously undescribed character combinations emphasize the complexity and plasticity of the early Palaeozoic aculiferan fauna, which follows the pattern of extreme disparity and convergences across Mollusca. Extant aplacophorans and chitons represent an anatomically and functionally depauperate sample of a diverse Palaeozoic group with a complex evolutionary history.

## Methods

### Physical–optical tomography

Both specimens were subjected to physical–optical tomography using the method detailed in ref. ^42^. Both specimens were serially ground at 20 μm intervals and photographed using a Leica digital microscope camera at a pixel resolution of 14.1 μm, in both cases as two separate datasets representing the two parts of the split concretion. Images were registered using SPIERSalign^43^. Raw physical–optical tomograms are provided for both specimens in the accompanying data^41^.

### Phase-contrast SXMT

Before physical–optical tomography, both specimens were scanned using phase-contrast SXMT with the PSICHÉ beamline of the SOLEIL synchrotron (Saint-Aubin, France) using a pink beam (63–69 keV), produced by filtering the synchrotron beam using 0.3 mm of tungsten and 1 mm of aluminium. The sample-to-detector propagation distance was 200 mm, and 3,000 projections were used. The exposure time was 30 ms per radiograph for a storage ring current of 16 mA in single bunch mode. The limited field of view available (12 mm × 3.6 mm) was extended horizontally by positioning the rotation axis off-centre, and extended vertically by recording a series of acquisitions with vertical movement of the sample. The volume (6.5 µm voxel size, no binning applied) was reconstructed from the combined radiographs using PyHST2 software^44^, with a Paganin phase retrieval algorithm^45^. The 32-bit greyscale float data of the reconstruction was converted to 8-bit for analysis, using the window of 0.1–0.6 for OUMNH PAL-C.29644 (*Punk*) and 0.1–0.7 for OUMNH PAL-C.36023 (*Emo*). Raw SXMT tomograms are provided for both specimens in the accompanying data^41^.

### Reconstructions

Phase-contrast SXMT does not typically perform well on specimens from the Herefordshire deposit, but for certain specimens it is effective^46^. In this study, the phase-contrast SXMT dataset proved superior to physical–optical tomography for OUMNH PAL-C.36023 (*Emo*) and was used to create the reconstructions in Fig. 2. For OUMNH PAL-C.29644 (*Punk*) the phase-contrast SXMT dataset lacked contrast and the reconstructions (Fig. 1) are based on the physical–optical dataset. Manual and semi-automatic segmentation was undertaken before reconstruction using the SPIERS suite^43^ to resolve fossil/matrix ambiguities and to classify portions of fossil (for example, separating spines from trunk). Spine segmentation utilized the SPIERSedit local contrast enhancement feature. The internal plate in *Punk ferox* was visualized using spline overlays (SPIERSedit ‘curves’ system), rather than by direct segmentation. Reconstructed models were generated and studied using SPIERview^43^, and publication images and stereo pairs (Figs. 1 and 2) were rendered using the cycles engine of Blender v.4.0 (ref. ^47^). Reconstructed models for both specimens are supplied^41^ in VAXML/STL format^48^.

### Phylogenetic analyses

The 51 taxa selected for this analysis include the species used in refs. ^1,15^ and a selection of more fossils and alternative living species used in refs. ^38^ and ^39^, plus the two new species described here (Supplementary Table 1). A character matrix was assembled based on previously published analyses for other Palaeozoic fossil lophotrochozoans, using the subset of characters relevant to molluscs taken from the most comprehensive previous analyses^38,39^, which in turn incorporated revised character sets based on earlier studies of fossil chitons^1,15^. We revised the character descriptions and added new characters in the light of previously undescribed morphologies observed in the new material, which resulted in a set of 108 characters (Supplementary Table 2). The coded matrix is provided in Supplementary Data 1 and 2 in MS Excel and NEXUS^49^ formats respectively.

Bayesian analyses were conducted using MrBayes^50,51^ v.3.2.7 via the CIPRES Science Gateway^52^ with two parallel runs on four chains in a temperature of 0.1, running indefinitely (100 million generations) but stopping automatically when the analysis reached posterior probability values below 0.01. The first 25% of results were discarded as burn-in. Commands are included in Supplementary Data 2; further analyses were run to test the influence of ordered multistate characters (command: ctype ordered/unordered) but this did not alter the resulting topology. Ordering commands are hence not included in the Supplementary Data. Further phylogenetic analyses using parsimony were conducted in TNT^53,54^, Willi Hennig Society edition, v.1.6. A traditional search was used with 5,000 random addition sequences, and Max Trees set to 9,999. Relevant multistate characters were set to ‘additive’ (=ordered) (Supplementary Data 2). Implied weights were not enabled. Results are shown in Extended Data Fig. 2. Finally, we reconstructed ancestral state conditions using parsimony based on the primary phylogenetic result (Fig. 3) using MESQUITE^55^.

### Reporting summary

Further information on research design is available in the Nature Portfolio Reporting Summary linked to this article.

## Online content

Any methods, additional references, Nature Portfolio reporting summaries, source data, extended data, supplementary information, acknowledgements, peer review information; details of author contributions and competing interests; and statements of data and code availability are available at 10.1038/s41586-024-08312-0.

## Supplementary information

Supplementary Information Supplementary Note 1 and Tables 1 and 2.

Reporting Summary

Supplementary Data 1 Phylogenetic matrix in MS Excel format.

Supplementary Data 2 Phylogenetic matrix in NEXUS format.

## Data Availability

All data required for assessing the conclusions are available online. The phylogenetic matrix is provided in Supplementary Data 1 and 2. Tomographic datasets and 3D models are available via the FigShare repository at 10.6084/m9.figshare.26340880 (ref. ^41^).
