# Supplementary material for: New Silurian aculiferan fossils reveal complex early history of Mollusca
Source: Nature. 2025 Jan 8;637(8046):631–6. doi: 10.1038/s41586-024-08312-0 (PMC11735398; doi:10.1038/s41586-024-08312-0)
Supplement: Supplementary file 1 — Supplementary Note 1 and Tables 1 and 2. [file 41586_2024_8312_MOESM1_ESM.pdf]

nature portfolio

https://doi.org/10.1038/s41586-024-08312-0

Supplementary information

New Silurian aculiferan fossils reveal complex early history of Mollusca

In the format provided by the authors and unedited

Supplementary Note 1

Expanded descriptions and discussions of preservational interpretations for *Punk ferox* and *Emo vorticaudum*.

Preservation of *Punk ferox*

Artefacts: There is minor data loss along the surface where the concretion split, the ventral surface in particular is partially effaced, and the absence of gills on the left-hand side after gill 4 is interpreted as an artefact. The distal part of the posterior ~30% of the spine array is also missing from reconstructions, as it fell outside the captured region of the physical-optical tomography dataset. This cut-off is visible in Fig. 1b. Unintended variation in tomogram-spacing is also reflected in the reconstruction, expressed as 'kinks' in the spines (Fig. 2b); the artefactual origin of these kinks is demonstrated by their consistent position, and the orientation of the changes in angle parallel to the tomographic plane. The main spine-set was reconstructed algorithmically (see methods), with manual interventions limited to per-tomogram compensations for variation in brightness caused by variation in photographic conditions. Discrimination between spines and unrelated dark 'speckles' in the matrix (ubiquitous in tomographic datasets from this locality, evident in Fig. 1o,p, for example) was not attempted within tomograms. While an 'island removal' filter was applied to the 3D model of the spines to remove the smallest and most numerous disconnected objects (see methods), spine reconstructions remain 'noisy'.

Extraneous material: The ventral surface of the specimen presented difficulties for our reconstruction methodology, which assumes a clear distinction between fossil and matrix and requires that voxels within a tomographic dataset can be assigned to one or other by algorithmic and/or manual segmentation. Here wispy dark material extends from the

undoubted fossil into the matrix over much of the ventral surface (Fig. 1o,p), often organized as one or more impersistent 'shells' surrounding the ventral region. We term this 'extraneous material', and have included it in reconstructions in a translucent form (Fig. 1b,j,u). This material is interpreted as primarily non-biological in origin, resulting from post-burial leakage of fluids from the decomposing body into the matrix, although the mechanisms involved are not well understood. The distinction between 'true' fossil and extraneous material is not always clear, as in places they blend into each other (e.g., Fig. 1o). Thus, the reconstructed boundary between fossil and extraneous material (where they are in contact) is an interpretation.

Internal structures: An elongate region of sediment is preserved internally on the left-ventral side (Fig. 1l-p). Sediment infiltration into cavities caused by decomposition is known from other Herefordshire fossils[56], and we interpret the sediment within the *Punk ferox* specimen similarly, as geopetal fill resulting from entry of sediment into the body cavity prior to 'freezing' of the matrix. A thin plate-like structure is preserved in the same region (Fig. 1l-p), which appears to have acted as a barrier to the percolation of sediment. This structure is only clearly preserved where sediment abuts it but is nonetheless distinct from adjacent fossil material (e.g., Fig. 1o), implying a distinctive taphonomic pathway and different composition from the rest of the fossil. It may have been at least partially mineralized. The plate is reconstructed using splines (see methods) and because of preservation and reconstruction methodology, its thickness and the position of its margins are minimal approximations; it may extend further and be thicker than indicated. The plate is of undoubted biological origin but is presumed to be displaced as it is preserved against the bottom margin of the fossil, the geopetal sediment-fill indicating way-up. We infer that there was an internal cavity prior to filling with sparry calcite[57] within which structures were prone to collapsing to the bottom (as

did the gut of *Acaenoplax*[19]). This must have occurred prior to the infiltration of sediment and is unlikely to have involved any significant displacement along the anterior-posterior axis of the specimen.

Inflation in region 2. The variable and asymmetric nature of the 'inflation' that characterizes the posterior of region 2 (see description below) suggests a taphonomic agent, perhaps relating to post-mortem release of gases in early decomposition, which may have been preferentially concentrated in this region. The relatively poor preservation of this region supports this interpretation, as does the location of the sediment fill.

Full description of *Punk ferox* holotype

The body is elongate, 21.0 mm in length. It is preserved with some lateral curvature (Fig. 1i-k) but none dorsoventrally (Fig. 1b,c); there is slight twisting (Fig. 1s). The width is constant at ~3.5-3.7 mm through 30-90% of the length. The specimen tapers gradually anteriorly to ~2mm before terminating in a rounded anterior margin (Fig. 1a,c,i-k,q,s,t). The posterior margin is sub-semicircular (Fig. 1k). A broad median ridge with a rounded top runs almost the full length of the dorsal surface but does not extend to either anterior or posterior termination (Fig. 1i,r,s,u). A low 'hump' is present immediately anterior to the mid-point (Fig. 1a,i,s) beyond which the ridge broadens (Fig. 1d-f,i) becoming gradually less prominent towards the anterior margin (Fig. 1c,e,i,s,u) with less steep lateral slopes (Fig. 1c,d). The ridge is narrow and steep-sided posteriorly (Fig. 1g-i). Its lateral margin is concave in cross-section, especially posteriorly (Fig. 1d-h), and an inflexion marks a sharp transition to the dorsolateral surface, which is subplanar or convex (Fig. 1d-h) in cross-section. The left and right dorsolateral surfaces curve to merge at the posterior margin (Fig. 1r).

The dorsolateral surfaces bear a dense array of long, thin spines flanking the median ridge (Fig. 1a,b,o,p,q,u). These are up to 6.5 mm long, subcircular in cross-section, and up to 0.1 mm in diameter (Fig. 1p). Spine length does not vary substantially except for some shorter spines near the anterior and posterior terminations (Fig. 1b). Spines arising from the dorsolateral surfaces closer to the trunk axis are longer than those arising more laterally (Fig. 1b,q,u). Local variation of ~25% in length is evident among well preserved spines (Fig. 1b); longer spines have a (weak) tendency to occur in 'bundles' (Fig. 1b). Except at the anterior, all spines are dorsally directed and weakly recurved towards the midline (Fig. 1a). There is a substantial gap between the left and right spine tips at the anterior of the trunk, but they become gradually closer posteriorly until they are adjacent (Fig. 1a). At the posterior termination, dorsally directed spines are shorter and arise medially from the convergence of the sub-lateral ridges (Fig. 1a,b). In the anterior ~20% of the trunk, variation in spine direction increases to generate a fanning out anteriorly and laterally (Fig. 1a,j,k,u). A small number of impersistently preserved shorter stout spines arise from the dorsal surface of the head near the anterior margin (Fig. 1a,j,q,u); spines are otherwise absent anterior to ~10% of length. All spines are essentially straight indicating that they are mineralized spicules rather than chitinous setae.

The ventral surface is not as well preserved as the dorsal (see Preservation above), suggesting that it represents a different tissue type. The junction between the dorsal and ventral surfaces of the trunk is consistently sharp (Fig. 1c,o,p,u); a sudden angular change of at least 90° is evident in cross-section (Fig. 1d-h). We interpret this junction as the boundary between a cuticular dorsal surface and a non-cuticular ventral surface.

The ventral surface broadly mirrors the dorsal surface in morphology, with a median ridge flanked by sub-horizontal lateral regions (Fig. 1b,e-h,k,o-r,t,u). We describe it in three regions (r1-3, Fig. 1c,k), anterior to posterior: region 1 extends to 8% of trunk length from the anterior; region 2 extends to ~50% of length; region 3 comprises the posterior 50%.

Region 1 is the 'head'. The ventral surface bears a sub-cylindrical 'boss' 1.5mm in diameter and 0.8 mm in height (Fig. 1c,d,j,k,q,s-u), interpreted as a buccal mass, and determining antero-posterior polarity. The margins are vertical and the ventral surface is weakly convex (Fig. 1d). The boss is subcircular in ventral view (Fig. 1t), the maximum width slightly posterior to the mid length. No openings are evident, although there are indications of a sub-semicircular anterior plate (Fig. 1t). Except posteriorly, the boss is surrounded by a subplanar to weakly concave surface ~0.3 mm wide (Fig. 1d,q,t), which undercuts the boss in places (Fig. 1d). The boss is elevated above the median ridge, which it abuts (Fig. 1t). The ventrolateral surfaces of region 1 are continuous with those of region 2 (Fig. 1q,t).

In region 2 the ventral median ridge is rounded (Fig. 1e,k), with steep subplanar lateral faces (Fig. 1e). The junction between ridge and subplanar ventrolateral surfaces is well defined anteriorly (Fig. 1e). The posterior portion of region 2 is characterized by asymmetrical 'inflation' of the median ridge, interpreted as a post-mortem artefact (see Preservation above). Here the ridge is broader, with shallower and more variable slopes (Fig. 1f). In this inflated portion a sharp junction between ridge and ventrolateral surfaces is absent, and in places there are no distinct ventrolateral surfaces (Fig. 1f). On the left side an anteriorly directed concavity (Fig. 1q) corresponds with the transition to the inflated portion but is absent on the right. The junction between regions 2 and 3 corresponds with the posteriad decrease in width of the dorsal median ridge at the rear of the dorsal hump (Fig. 1c,i,k)

In region 3 the ventral median ridge is high and V-shaped in cross section (Fig. 1g,h). The sharp peak is typically preserved bending towards the right side (Fig. 1k) and is continuous with the extraneous material (Fig. 1j); it may thus represent a taphonomic artefact or be taphonomically exaggerated. The ventrolateral surfaces are subplanar to weakly concave, and their junction with the median ridge is sharp and well defined. At the extreme posterior the ventrolateral surfaces and median ridge merge (Fig. 1j,k). The ventrolateral surfaces bear variably preserved short sub-conical projections adjacent to the median ridge, interpreted as gills on the basis of their position and serial repetition (Fig. 1b,c,h,j,k,r,u). The gills are approximately 0.2 mm in diameter, up to 0.3 mm in height, and are 0.3-0.4 mm apart. Inconsistent preservation and minor data loss associated with the split between the two parts of the concretion (Fig. 1c) militate against an accurate count; we estimate there to be ~25 on each side.

A thin internal plate (Fig. 1l-p) is present to the right of the ventral median ridge in the posterior of region 2, associated with a partial geopetal sediment fill that indicates a post-burial way-up orientation towards the left side (Fig. 1m,o,p). In places the plate is coincident with preserved edge of the fossil (fig. 1p), but elsewhere it lies inside the fossil, i.e. within the body cavity (Fig. 1o). This plate is compositionally differentiated and may have been mineralized. It has moved from an unknown original position, but this displacement likely lacked a significant anterior-posterior component. The reconstructed extent of this plate (Fig. 1l-n) is a minimal estimate, but in its preserved and reconstructed position it is restricted to region 2. See Preservation above for further interpretation of this structure and associated sediment fill.

Preservation of *Emo vorticaudum*

Artefacts: There is minor data-loss along the surface where the concretion split as in *Punk ferox*, and the distal part of the spine array is missing from reconstructions of the mid-body (Fig. 2b) as it fell outside the captured region of the dataset. Reconstructions of the spine-set remain noisy for the same reasons as given above for *Punk ferox*. Reconstructions show a dorsal 'bald patch' towards the posterior of the trunk (Fig. 2a,p); no spines are visible in this region in tomograms from the synchrotron dataset (Extended Data Fig. 1c; compare Extended Data Fig. 1d) and the adjacent matrix is unusually dark and contains fine cracks (cr; Extended Data Fig. 1c). The physical-optical dataset displays a lighter (chemically reduced) area in this region of matrix (Fig. 2l, Extended Data Fig. 1a) within which evidence of spines can discerned (sp; Extended Data Fig. 1a). Thus, the apparent absence of spines in the bald patch reflects this lighter area, which is inferred to have resulted in reduced X-ray contrast between spines and matrix in the synchrotron tomograms. Such lighter areas are common in Herefordshire fossils[54], although their mechanism of formation is unclear. The apparent break on the dorsal surface in the same region is also an artefact of this lighter area, as the cuticle is continuous in physical-optical tomograms (Fig. 2l, Extended Data Fig.1a). A similar consideration applies to breaks on the ventral surface (Extended Data Fig. 1a,b).

Internal structures: Material comprising the fossil is differentiated into two phases; in optical tomograms these appear as (a) sparry calcite crystals, and (b) dark fine-grained material. In synchrotron tomograms, the former is more X-ray dense than the matrix, but the latter is indistinguishable from the matrix. Thus, the dark material appears as cavities in synchrotron tomograms and resulting reconstructions (visible through apparent cuticular breaks in e.g., Fig. 2p). The composition of the dark material is unknown, but it resembles the soft-tissue phase of the Herefordshire echinoderm *Bdellacoma*[58]. Differences in X-ray absorption

between this and the sparry calcite imply some degree of chemical differentiation. The arrangement of this material is at least in part bilaterally symmetrical, and it may represent internal features. However, we cannot exclude the possibility of preservational artefacts.

Full description of *Emo vorticaudum* holotype

The specimen is elongate, 17.1 mm long (unfolded), preserved with a ~45° dorsoventral flexure near the mid length (Fig. 2b,c). It is 3.5 mm in maximum width (at the midpoint), and 2.5 mm in maximum height (at ~25% of length from the anterior). The body is divided into four regions (r1-4 anterior to posterior, Fig. 2c,j,k). Region 1 is a short 'head', 1.1 mm long. Region 2 is a 'neck', 2 mm in length. Region 3 is the main trunk, 11.5 mm in length. Region 4 is a 'tail', 2.5 mm in length.

Region 1 (the 'head') bears two small valves, Valve I on the anterior surface, and Valve II on the dorsal surface (Fig. 2a,k,r-t). The valves are in near contact, with an angle of ~70° between them (Fig. 2t). Valve I is sub-ovoid in outline (Fig. 2r), 1.35 mm wide and 1.2 mm long. The dorsal surface comprises two distinct lateral areas, weakly concave, subtending an angle of ~120° and meeting at a rounded median ridge with an apex at about 75% of valve length from the anterior (Fig. 2r). Valve II is similar to Valve 1, 1.5 mm wide and 1.22 mm long, but the anterior margin protrudes farther (Fig. 2s). The apex is sub-central, slightly anterior to the midpoint, and the lateral surfaces subtend an angle of ~100° (Fig. 2d). Valve II preserves a weak growth line close to the margin (Fig. 2t); neither valve preserves any other surface feature. Ventral valve-surfaces are unknown. The valves cover the dorsal and anterior surfaces of the head almost entirely (Fig. 2r-t). The lateral surface of region 1 extends ventrally into a raised sub-oval (long axis anterior-posterior) central region. A weak elongate median hollow (Fig. 2j,m,t) is interpreted as an oral opening. Region 1 is semi-circular in

cross section, capped by Valve II (Fig. 2d). Immediately posterior to the raised ventral region a deep transverse groove marks the junction with region 2 (Fig. 2f); this is positioned approximately in line with the apex of Valve II, which overlaps the anteriormost part of region 2.

The ventral surface of the anterior of region 2 is anteriorly co-planar with that of region 1 (Fig. 2j,m,t). Near the middle of region 2 the trunk expands relatively abruptly in a ventral direction (Fig. 2j,m). The ventral, lateral, and dorsal surfaces are continuous and curve smoothly into each other without consistent sharp junctions (Fig. 2e,f,m); the lateral surfaces are likewise continuous with those of regions 1 and 3, and the ventral surface with that of region 3 (Fig. 2b,j,m). Region 2 is sub-circular in cross-section anteriorly (Fig. 2e), becomes increasingly sub-oval posteriorly (Fig. 2f) as the dorsal surface is elevated into an elongate dorsal 'hump' (Fig. 2c,f) with a rounded peak. The sides of region 2 are straight and near parallel, tapering very slightly anteriorly (Fig. 2j,k).

The boundary between regions 2 and 3 is gradational. Region 3 is characterized by lateral rolls, discrete thickenings of the lateral margin separated from the dorsal and ventral surfaces by clear breaks of slope (Fig. 2g,h,j,k,m), which merge anteriorly into the flanks of region 1. The rolls extend around the posterior of the trunk to join medially (Fig. 2j,m) beneath the attachment of region 4 (Fig. 2k). The dorsal surface of region 3 is evenly convex (Fig. 2g,h). The ventral surface is flattened medially in the anterior part of region 3 (Fig. 2g,m), becoming evenly convex posteriorly (Fig. 2h). The cross-section becomes less convex posteriorly as the height of the trunk decreases (compare Fig. 2g,h). The ventral surface of the trunk bears at least two transverse ridges anterior to the fold (Fig. 2b) which we interpret as in vivo deformation resulting from the folded posture. Posteriorly, the ventral surface displays a

weakly pustulose texture. There is no sharp junction between ventral and dorsal surfaces, and their preservation is not differentiated. Further, the ventral surface of region 3 is continuous with that of region 2, which is itself continuous with lateral and dorsal surfaces. This leads us to interpret both dorsal and ventral surfaces as cuticular in nature; there is no foot.

Regions 2 and 3 bear an array of spines, up to 3.5 mm long and 0.08 mm in diameter, that form a dorsal and lateral scleritome. The longest spines arise from the lateral rolls. Spines are absent on the ventral surface and the lateral surfaces of region 2. Evidence of short spines posterior of Valve II suggests that they are present on the dorsal surface of region 2 immediately posterior to this valve. The 'bald patch' in the posterior dorsal scleritome (Fig. 2a,p) is interpreted as a preservational artefact (see Preservation above). Larger dorsomedial spines are present at the extreme trunk posterior and may represent a dorsal expression of the merged lateral rolls.

Spines arising from the ventral part of the lateral rolls are straight, directed laterally and slightly posteriorly (e.g., Fig. 2j, mid-trunk). The spines in a more dorsal position on the lateral rolls are directed dorsolaterally. Those further toward the trunk axis become weakly recurved medially (Fig. 2p). Finer spines arising from the dorsal trunk surface are also directed dorsomedially and recurved, such that they 'criss-cross' medially (Fig. 2a,p). The spines in region 2 are shorter and finer and show a more pronounced posterior direction (Fig. 2j). Large spines are almost absent where the lateral rolls converge at the posterior end of the trunk. Those present include a prominent medial spine directed posteroventrally at ~45° from the ventral surface of the roll (vs; Fig. 2b,j,m,p), a pair of long spines directed laterally and slightly posteriorly (ls; Fig. 2a,j,m,p), and a single spine, directed posterolaterally, on the left

side (ss; Fig. 2m,p). All spines are essentially straight and we interpret them as mineralized spicules rather than chitinous setae.

Region 4 is sub-circular in cross section, weakly compressed dorsoventrally (Fig. 2I). It tapers evenly from ~1.5 mm in width anteriorly to 1 mm posteriorly (Fig. 2c,k). The long axis is oriented upwards at an angle ~30° to the posterior of the trunk (Fig. 2c,k) and slightly laterally; this may reflect *in vivo* flexibility. There is a ridge around the circumference of region 4 at its anterior extremity (br; Fig. 2c,j,k,q). The distal (posterior) tip is produced into a set of poorly preserved posteriorly directed stubby protuberances, interpreted as respiratory organs (Fig. 2q). The region is surrounded by an array of spines (Fig. 2a,b,j,p) arising from a section ~0.5 mm long immediately posterior to the basal ridge (smooth portion of r4 in Fig. 2q). The spines are straight, up to 2 mm long, and are directed posteriorly with a ~20° twist. They arise at a very low angle and co-terminate ~0.3 mm posterior of the respiratory projections to form a spine-enclosed posteriorly-opening chamber (Fig. 2p).

Four separate structures (i1-4, Fig. 2g,n,o) are preserved in dark material within the body (Fig. 2l) (see Preservation section). Internal structure 1 (i1, Fig. 2g,l,n,o) is positioned ventromedially in region 3, largely anterior to the dorsoventral fold, forming a thin incomplete layer inside the ventral cuticle. It is sub-lunate in ventral view, tapering anteriorly, and extending asymmetrically into posterolateral arms which reach the margins of the lateral rolls. Internal structure 2 (i2, Fig. 2h,l,n,o) is positioned posterodorsally in the trunk. It is also asymmetrically sub-lunate, tapering posteriorly, with arms extending antero-laterally. Structure 2 becomes thicker as it tapers (Fig. 2l,n). Structure 3 (i3, Fig. 2i,l,n,o) is sub-cylindrical and largely confined to region 4, where it lies ventrolaterally to the midline (Fig. 2i) but not in contact with the surface of the body. It is 0.2 mm in diameter posteriorly but

flares anteriorly, extending into the trunk as two impersistently preserved lateral arms. The margins of the sub-cylindrical region are more clearly preserved than the interior, resulting in a hollow reconstruction. Structure 4 (i4, Fig. 4l,n,o) is restricted to the posterior 1 mm of region 4 as an incomplete layer near the lateral and posterior margins. These internal structures are difficult to interpret: structure 3 may (at least in part) represent the gut, and structure 4 was likely part of the respiratory system.

Additional References

56. Sutton, M. D., Briggs, D. E. G., Siveter, David J. & Siveter, Derek J. A three-dimensionally preserved fossil polychaete worm from the Silurian of Herefordshire, England. *Proc. R. Soc. B* **268**, 2355-2363 (2001).

57. Orr, P. J., Briggs, D. E. G., Siveter, David J. & Siveter, Derek J. Three-dimensional preservation of a non-biomineralized arthropod in concretions in Silurian volcaniclastic rocks from Herefordshire, England. *Jl Geol. Soc.* **157**, 173-186 (2000).

58. Sutton, M. D., Briggs, D. E. G., Siveter, David J., Siveter, Derek J. & Gladwell, D. J. A starfish with three-dimensionally preserved soft parts from the Silurian of England. *Proc. R. Soc. B* **272**, 1001-1006 (2005).

Supplementary Table 1

Taxa included in the present analysis and citations to previous studies.

Clade	Terminal	Previous analyses
Mollusca		
	Odontogriphus	1,15,38,39
	Wiwaxia	1,15,38,39
	Shishania	39
Mollusca: Aculifera		
	Acaenoplax	1,15,38,39
	Calvapilosa	38,39
	Emo	This study
	Enetoplax	1,15,38,39
	Halkieria	1,15,38,39
	Heloplax	1,15,38,39
	Orthrozanclus	1,15,38,39
	Punk	This study
Mollusca: Aculifera: Aplacophora		
	Chaetoderma	1,15,38,39
	Genitoconia	38,39
Mollusca: Aculifera: Polyplacophora: Multiplacophora		
	Echinochiton	1,15,38,39
	Polysacos	1,15,38,39
	Protobalanus	15,38,39
	Strobilepis	1,15,38,39
Mollusca: Aculifera: Polyplacophora: Neoloricata		
	Acutichiton	1,15,38,39
	Chaetopleura	1,15,38,39
	Glaphurochiton	1,15,38,39
	Gryphochiton	1,15,38,39
	Leptochiton	1,15,38,39

	Ochmazochiton	1,15,38,39
	Pedanochiton	1,15,38,39
	Tonicella	38
Mollusca: Aculifera: Polyplacophora: Paleoloricata		
	Kulindroplax	15,38,39
	Matthevia	1,15,38,39
	Phthipodochiton	1,15,38,39
	Robustum	1,15,38,39
	Septemchiton	1,15,38,39
Mollusca: Conchifera		
	Pelagiella	38,39
	Yochelcionella	38,39
Mollusca: Conchifera: Bivalvia		
	Anadara	38,39
	Babinka	15,38,39
	Fordilla	15,38,39
	Modiolus	38,39
	Nucula	38,39
	Pojetaia	15,38,39
Mollusca: Conchifera: Gastropoda		
	Buccinum	38,39
	Crepidula	38,39
	Lottia	38,39
	Neptunea	38,39
Mollusca: Conchifera: Monoplacophora		
	Neopilina	1,15
Mollusca: Conchifera: Rostroconchia		
	Conocardium	38,39
Mollusca: Conchifera: Scaphopoda		
	Antalis	38,39
Outgroups: Annelida		
	Serpulidae	38,39
Outgroups: Brachiopoda		

	Lingula	1,15,38,39
	Mickwitzia	1,15,38,39
	Micrina	38,39
	Novocrania	1,15,38,39
	Terebratulina	38,39

Supplementary Table 2

Characters used in present phylogenetic analysis. Source column indicates the original source where the character was used in earlier analyses, and Comments column where the character interpretation differs here from prior studies. Multistate characters marked * are treated as ordered.

#	Character	Coding	Source : character number	Comments
1	Body shape	0 dorsoventrally flattened<br>1 vermiform<br>2 laterally compressed	48: 101	Revised coding
2	Posterior elongation	0 Absent / 1 Present	48: 104	
3	Anterior elongation	0 Absent / 1 Present	48: 105	
4	Torsion	0 Absent / 1 Present	48: 106	
5	Longitudinal muscle bands	0 Absent / 1 Present	48: 107	
6	Circular muscles	0 Absent / 1 Present	48: 108	
7	Co-ordinated serial repetition of multiple body parts	0 Absent / 1 Present	48: 112	
8	Mantle cavity restricted posterior	0 Absent / 1 Present	48: 131	
9	Ctenidia	0 Absent / 1 Present	48: 132	
10	Serial ctenidia	0 Absent / 1 Present	48: 133	
11*	Pairs of ctenidia	0 one pair<br>1 three or four pairs<br>2 five pairs<br>3 eight or more pairs	48: 134	
12	Ventral respiratory cavity	0 Absent / 1 Present	48: 135	
13	Ventral ctenidia posterior only	0 Absent / 1 Present	48: 137	
14	Gill morphology	0 unipectinate<br>1 bipectiate	48: 138	
15	Unpaired gills	0 Absent / 1 Present	48: 139	
16*	Muscular foot	0 Absent<br>1 Present<br>2 Pedal groove	48: 144	Revised character and coding
17	Foot modified for digging	0 Absent / 1 Present	48: 145	
18	Foot reduced or secondarily absent	0 Absent / 1 Present		New
19	Paleae or flattened chaetae/sclerites, including the flat sclerites of aculiferan molluscs	0 Absent / 1 Present	48: 197	

#	Character	Coding	Source : character number	Comments
20	Stalked chaetae or sclerites	0 Absent / 1 Present	48: 198	
21	Hollow sclerites or chaetae	0 Absent / 1 Present	48: 199	
22	Mineralized chaetae	0 Absent / 1 Present	39: 200	
23	Ligaments	0 Absent / 1 Present	39: 212	
24	Primary body axis	0 typical anterior-posterior axis<br>1 U-shape from ano-pedal flexion	39: 255	Revised character and coding
25	Buccal organ or pharynx	0 Absent / 1 Present	39: 261	Revised character and coding
26	Buccal organ position	0 terminal<br>1 ventral	39: 262	Revised character and coding
27	Radula	0 Absent / 1 Present	39: 283	
28	Number of tooth rows in radula	0 few rows<br>1 many rows<br>2 a single row	39: 284	
29	Shell plates or sclerites with continual accretionary growth	0 Absent / 1 Present	39: 292	
30	Calcium phosphate biomineralization	0 Absent / 1 Present	39: 347	
31	Calcium carbonate biomineralization	0 Absent / 1 Present	39: 348	Revised coding
32	Calcium carbonate polymorph	0 Calcite<br>1 Aragonite	39: 349	
33	Shell deposition with alternation of dense and porous layers	0 Absent / 1 Present	39: 351	
34	Shell microstructure with fibrous bundles	0 Absent / 1 Present	39: 352	
35	Tabular laminar secondary layer	0 Absent / 1 Present	39: 353	
36	Laminated or stratiform shell microstructure	0 Absent / 1 Present	39: 354	
37	Shell or biomineralized parts, growth by marginal accretion	0 Absent<br>1 Present	39: 366	
38	Scleritome basic components	0 shell plates<br>1 sclerites (many small mineralized components)<br>2 combination of plate(s) and smaller sclerites		New
39	How many shell plates?	0 absent<br>1 = 1<br>2 = 2<br>3 = more than 2		New

#	Character	Coding	Source : character number	Comments
40	Zones in transverse section at mid-length of animal, including sclerites and valves (counted for one lateral half):	0 one<br>1 two<br>2 three<br>3 four or more	15: 36	
41	Serial repetition of self-similar sclerites or shell plates	0 Absent / 1 Present	39: 377	
42	Marginal setal fringe at aperture	0 Absent / 1 Present	39: 378	
43	Serial muscle scars on shell	0 Absent / 1 Present	39: 380	
44	Byssus	0 Absent / 1 Present	39: 381	
45	Byssal gape	0 Absent / 1 Present	39: 382	
46	Sclerites or shells enveloping the body	0 Absent / 1 Present	39: 435	
47	Pegma	0 Absent / 1 Present	39: 437	
48	Anterior placed head plate	0 Absent / 1 Present	39: 440	
49	Posteriorly placed tail valve	0 Absent / 1 Present	39: 441	Revised coding
50	Serially repeated median shell fields (in adult or embryo)	0 Absent / 1 Present	39: 442	
51	Intermediate shell plates	0 Absent / 1 Present	39: 443	
52	Differentiated shell fields in intermediate plates	0 Absent / 1 Present	39: 444	
53	Lateral division of intermediate shell plates	0 Absent / 1 Present	39: 445	
54*	Number of non terminal shell valves	0 none<br>1 one<br>2 five or six	39: 446	Revised character and coding
55	Shell pores	0 Absent / 1 Present	39: 447	
56	Shell plate with aesthete canal system	0 Absent / 1 Present	39: 449	
57	Sclerites with pores	0 Absent / 1 Present	39: 448	
58	Tunnels in shell valve(s)	0 Absent / 1 Present	39: 450	
59	Lateral tunnels flanking median	0 Absent / 1 Present	15: 12	
60	Tunnels at which end of valve	0 posterior<br>1 anterior	15: 13	
61	Articulamentum layer of shell valve(s)	0 Absent / 1 Present	39: 452	
62	Insertion plates formed by articulamentum	0 Absent / 1 Present	39: 454	
63	Slitted insertion plates	0 Absent / 1 Present	39: 455	
64	Pectinated insertion plates	0 Absent / 1 Present	39: 456	

#	Character	Coding	Source : character number	Comments
65	Length of apical area of non-terminal valve	0 less than 10% valve length<br>1 more than 10% of valve length	39: 458	
66	Growth style anterior valve	0 holoperipheral<br>1 mixoperipheral or hemiperipheral	39: 459	
67	Primary growth margin of anterior valve	0 anterior<br>1 posterior	39: 460	
68*	Length /width ratio of anterior plate	0 elongate<br>1 equal to subequal<br>2 transverse	39: 461	
69*	Posterior margin of anterior valve	0 concave<br>1 sub linear<br>2 convex	39: 462	
70	Growth pattern of tail valve	0 holoperipheral<br>1 mixoperipheral or hemiperipheral	39: 463	
71	Length /width ratio of tail valve	0 elongate<br>1 equal to subequal<br>2 transverse	39: 464	
72*	Anterior margin of tail valve	0 concave<br>1 sublinear<br>2 convex	39: 465	
73	Non-terminal valve growth style	0 holoperipheral<br>1 mixoperipheral or hemiperipheral	39: 466	
74	Length /width ratio non terminal valve(s)	0 equal or subequal<br>1 elongate	39: 467	Revised character and coding
75*	Anterior margin non terminal valve(s)	0 concave<br>1 linear<br>2 convex	39: 468	
76*	Posterior margin non-terminal valve(s)	0 concave<br>1 linear<br>2 convex	39: 469	
77	Apical angle non-terminal valve(s)	0 less 90 degrees<br>1 more 90 degrees	39: 470	
78	Coiled shell	0 Absent / 1 Present	39: 471	
79	Siphonal notch	0 Absent / 1 Present	39: 475	
80	Ventral suture	0 Absent / 1 Present	39: 476	
81	Left and right shells with hinge	0 Absent / 1 Present	39: 477	
82	Curved ventral shell margin (as in bivalves)	0 Absent / 1 Present	39: 478	Revised coding
83	Posterior mantle opening in shell	0 Absent / 1 Present	39: 479	
84	Operculum	0 Absent / 1 Present	39: 480	Revised coding

#	Character	Coding	Source : character number	Comments
85	Granular/spinose sculpture/ornament on dorsal surface of any valves	0 Absent / 1 Present	15: 8	
86	Concentric ornament	0 Absent (or growth lines only)<br>1 Present	15: 9	
87	Radial ornament	0 Absent / 1 Present	15: 10	
88	Valve series of at least two adjacent valves	0 Absent / 1 Present	15: 22	
89	Overlap of valve series:	0 none<br>1 In contact in at least part of series (e.g. *Cryptoplax*);<br>2 with overlap.	15: 23	
90	Side slopes of non-terminal valves	0 concave;<br>1 straight(ish);<br>2 convex.	15: 31	
91	Crystalline or apparently monocrystalline spicules	0 Absent / 1 Present	15: 32	
92	Hollow organic sclerites (e.g. *Wiwaxia*)	0 Absent / 1 Present	15: 33	
93	Hollow lateral spines (e.g. *Echinochiton*)	0 Absent / 1 Present	15: 34	
94	Solid lumpy mineralized sclerites (e.g. *Acanthopleura*)	0 Absent / 1 Present	15: 35	
95	Ventral surface with cuticle-covered sole	0 Absent / 1 Present		New
96	Scleritome with additional, sparse, outwardly directed spines	0 Absent / 1 Present		New
97	Scleritome with anterior-posterior differential patterning,	0 uniform<br>1 three zones		New
98	Mouth complex	0 simple mouth<br>1 oral shield or disc (as in monoplacohora, polyplacophora)		New
99*	Molluscan shell form	0 no<br>1 solid shell(s) without additional consistent armature<br>2 scleritome potentially including serialized shells		New
100	Evidence of scleritome embedded in cuticle	0 Absent / 1 Present		New
101	Scleritome including elongate spines	0 Absent / 1 Present		New
102	Posterior tuft	0 Absent / 1 Present		New

#	Character	Coding	Source : character number	Comments
103	Larval shell (protoconch, prodissoconch) as basis for adult shell accretionary growth	0 Absent / 1 Present		New
104	Gut coiled or folded (not including AP flexion)	0 Absent / 1 Present	39: 256	New character framing
105	Elongation of dorsal-ventral body axis and nervous system	0 Absent / 1 Present	39: 402	New character framing
106	Repeated shell fields in embryo	0 Absent / 1 Present		New
107	Evidence of cephalization / cephalized senses	0 Absent / 1 Present		New
108	Mineralized shell(s) can protect the whole body	0 No<br>1 Yes		New
