# Supplementary material for: New Silurian aculiferan fossils reveal complex early history of Mollusca
Source: Nature. 2025 Jan 8;637(8046):631–6. doi: 10.1038/s41586-024-08312-0 (PMC11735398; doi:10.1038/s41586-024-08312-0)
Supplement: Supplementary file 2 — Reporting Summary [file 41586_2024_8312_MOESM2_ESM.pdf]

nature portfolio

Corresponding author(s): Mark Sutton

Last updated by author(s): Sep 16, 2024

Reporting Summary

Nature Portfolio wishes to improve the reproducibility of the work that we publish. This form provides structure for consistency and transparency in reporting. For further information on Nature Portfolio policies, see our Editorial Policies and the Editorial Policy Checklist.

Statistics

For all statistical analyses, confirm that the following items are present in the figure legend, table legend, main text, or Methods section.

n/a	Confirmed	
☒	☐	The exact sample size (*n*) for each experimental group/condition, given as a discrete number and unit of measurement
☒	☐	A statement on whether measurements were taken from distinct samples or whether the same sample was measured repeatedly
☒	☐	The statistical test(s) used AND whether they are one- or two-sided<br>*Only common tests should be described solely by name; describe more complex techniques in the Methods section.*
☒	☐	A description of all covariates tested
☒	☐	A description of any assumptions or corrections, such as tests of normality and adjustment for multiple comparisons
☒	☐	A full description of the statistical parameters including central tendency (e.g. means) or other basic estimates (e.g. regression coefficient) AND variation (e.g. standard deviation) or associated estimates of uncertainty (e.g. confidence intervals)
☒	☐	For null hypothesis testing, the test statistic (e.g. *F*, *t*, *r*) with confidence intervals, effect sizes, degrees of freedom and *P* value noted<br>*Give P values as exact values whenever suitable.*
☐	☒	For Bayesian analysis, information on the choice of priors and Markov chain Monte Carlo settings
☒	☐	For hierarchical and complex designs, identification of the appropriate level for tests and full reporting of outcomes
☒	☐	Estimates of effect sizes (e.g. Cohen's *d*, Pearson's *r*), indicating how they were calculated

Our web collection on statistics for biologists contains articles on many of the points above.

Software and code

Policy information about availability of computer code

Data collection	N/A
Data analysis	These are all cited in methods:<br>Spencer, A. R. T., Garwood, R. J. & Sutton, M. D. SPIERS, version 3.1.0. palaeoware/SPIERS (2020). https://doi.org/10.5281/zenodo.3859623.<br>Zhang, C., Huelsenbeck, J., van der Mark, P., Ronquist, F. & Teslenko, M. MrBayes: Bayesian Inference of Phylogeny, version 3.2.5 (2015). https://github.com/NBISweden/MrBayes<br>Goloboff, P. A., Farris, J. S. & Nixon, K. C. TNT (Willi Hennig Society edition), version 1.6. (2024). https://www.lillo.org.ar/phylogeny/tnt/.<br>Miller, M. A., Pfeiffer, W., & Schwartz, T. Creating the CIPRES Science Gateway for inference of large phylogenetic trees. Proceedings of the Gateway Computing Environments Workshop (GCE), 14 Nov. 2010, New Orleans. 1-8 (2010).<br>Maddison, W. P. & Maddison, D. R. Mesquite: a modular system for evolutionary analysis, version 3.81 (2023). http://www.mesquiteproject.org.

For manuscripts utilizing custom algorithms or software that are central to the research but not yet described in published literature, software must be made available to editors and reviewers. We strongly encourage code deposition in a community repository (e.g. GitHub). See the Nature Portfolio guidelines for submitting code & software for further information.

Data

Policy information about availability of data

All manuscripts must include a data availability statement. This statement should provide the following information, where applicable:

- Accession codes, unique identifiers, or web links for publicly available datasets
- A description of any restrictions on data availability
- For clinical datasets or third party data, please ensure that the statement adheres to our policy

All data required for assessing the conclusions are available online. The phylogenetic matrix is provided in Supplementary Data 1 and 2. Tomographic datasets and 3D models are provided in the accompanying FigShare[41] repository.
[41] is a reference to the figshare repository accompanying the submission.

Research involving human participants, their data, or biological material

Policy information about studies with human participants or human data. See also policy information about sex, gender (identity/presentation), and sexual orientation and race, ethnicity and racism.

Reporting on sex and gender	N/A
Reporting on race, ethnicity, or other socially relevant groupings	N/A
Population characteristics	N/A
Recruitment	N/A
Ethics oversight	N/A

Note that full information on the approval of the study protocol must also be provided in the manuscript.

Field-specific reporting

Please select the one below that is the best fit for your research. If you are not sure, read the appropriate sections before making your selection.

[x] Life sciences [ ] Behavioural & social sciences [ ] Ecological, evolutionary & environmental sciences

For a reference copy of the document with all sections, see nature.com/documents/nr-reporting-summary-flat.pdf

Life sciences study design

All studies must disclose on these points even when the disclosure is negative.

Sample size	Specimens are 'singletons' - there is no sampling concept. Phylogenetic datasets use taxon-sets that follow previous publications.
Data exclusions	N/A
Replication	N/A
Randomization	N/A
Blinding	N/A

Reporting for specific materials, systems and methods

We require information from authors about some types of materials, experimental systems and methods used in many studies. Here, indicate whether each material, system or method listed is relevant to your study. If you are not sure if a list item applies to your research, read the appropriate section before selecting a response.

Materials & experimental systems

n/a	Involved in the study
☒	☐ Antibodies
☒	☐ Eukaryotic cell lines
☐	☒ Palaeontology and archaeology
☒	☐ Animals and other organisms
☒	☐ Clinical data
☒	☐ Dual use research of concern
☒	☐ Plants

Methods

n/a	Involved in the study
☒	☐ ChIP-seq
☒	☐ Flow cytometry
☒	☐ MRI-based neuroimaging

Palaeontology and Archaeology

Specimen provenance	Herefordshire, UK. No permits required.
Specimen deposition	Oxford University Museum of Natural History
Dating methods	Long-established stratigraphic age of unit. Note that I have ticked the 'raw and calibrated' option to satisfy the form, but this is not really relevant to Palaeozoic fossils

☒ Tick this box to confirm that the raw and calibrated dates are available in the paper or in Supplementary Information.

Ethics oversight	No ethical approval required for UK invertebrate specimens.

Note that full information on the approval of the study protocol must also be provided in the manuscript.

Plants

Seed stocks	N/A
Novel plant genotypes	N/A
Authentication	N/A
